# Research on Over-the-Horizon Perception Distance Division of Optical Fiber Communication Based on Intelligent Roadways

**DOI:** 10.3390/s24010276

**Published:** 2024-01-03

**Authors:** Xin An, Baigen Cai, Linguo Chai

**Affiliations:** School of Electronic and Information Engineering, Beijing Jiaotong University, Beijing 100044, China

**Keywords:** over the horizon perception, distance division, optical fiber communication, streaming perception, intelligent roadway, road classification

## Abstract

With the construction and application of more and more intelligent networking demonstration projects, a large number of advanced roadside digital infrastructures are deployed on both sides of the intelligent road. These devices sense the road situation in real time through algorithms and transmit it to edge computing units and cloud control platforms through high-speed optical fiber transmission networks. This article proposes a cloud edge terminal architecture system based on cloud edge cooperation, as well as a data exchange protocol for cloud control basic platforms. The over-the-horizon scene division and optical fiber network communication model are verified by deploying intelligent roadside devices on the intelligent highway. At the same time, this article uses the optical fiber network communication algorithm and ModelScope large model to model inference on real-time video data. The actual data results show that the StreamYOLO (Stream You Only Look Once) model can use the Streaming Perception method to detect and continuously track target vehicles in real-time videos. Finally, the method proposed in this article was experimentally validated in an actual smart highway digital infrastructure construction project. The experimental results demonstrate the high application value and promotion prospects of the fiber optic network in the division of over the horizon perception distance in intelligent roadways construction.

## 1. Introduction

Intelligent roadways refer to roads that are interconnected with vehicles, traffic lights, sensors and other equipment through various advanced technological means to achieve intelligent management and operation. Among them, wireless communication technology, Internet of Things technology, cloud computing technology, etc., can be applied in the construction of intelligent roads. Through the Internet of Things technology, intelligent roadways can be monitored in real time and data can be collected to detect and solve road traffic problems promptly. Through cloud computing technology, the collected data can be processed and analyzed to provide more accurate traffic management and services.

Optical networks are a type of network that uses light waves as a carrier and utilizes the physical characteristics of light waves for data transmission [1]. They have the advantages of high speed, large bandwidth, and low loss, and are one of the important technologies for building modern communication networks. Optical networks also have important applications in intelligent roadway construction. At the same time, optical networks can also provide more stable and reliable communication connections for intelligent vehicles to improve their driving safety and efficiency [2].

Intelligent intersections mainly include signal lights, traffic signal systems, video surveillance systems, traffic bayonets, electronic police, microwave radar, and other facilities and equipment. Due to differences in the construction of the main components, these devices and systems were mostly built at different times, which leads to a lack of information and data sharing between systems and equipment [3]. In terms of operation and maintenance, the resource utilization rate of the hardware system is not high. The OTA (Over-the-Air Technology, OTA) remote automatic upgrade function cannot be adopted, and SDN (Software Defined Network, SDN) technology cannot be adopted to achieve the iterative upgrade function of the system and functions. The scalability is not strong [4].

Fiber optic networks have a wide range of applications in intelligent road construction, including information acquisition and transmission, intelligent upgrading and transformation, intelligent traffic signal control, connection of digital infrastructure equipment, and high-speed transmission of large-capacity video data and point cloud data.

Through analyzing a large number of smart road project construction practices, it has been found that in the construction of smart roads, a large number of digital infrastructure devices, such as cameras, radars, meteorological sensors, etc., are deployed on the roadside. These devices generate a large amount of image and point cloud information during operation. These massive amounts of information need to be processed at the collection side, some need to be transmitted to the edge cloud for processing, and some need to be transmitted to the cloud control platform for processing. Therefore, this article proposes a fiber optic transmission network architecture based on edge cloud architecture.

This article is mainly divided into five sections. Section 1 serves as the introduction, providing an overview of the research background and the current development status. In Section 2, we address the study motivation and objectives of this article. Section 3 explores the architecture of fiber optic transmission networks under cloud edge architecture, such as the over-the-horizon scenario division and optical fiber communication based on cloud edge architecture, interactive multiple model algorithms for highway vehicle target tracking, and intelligent highway vehicle target detection. In Section 4, we delve into the intelligent highway deployment architecture. Finally, in Section 5, we draw our conclusions and provide a prospective outlook for the entire article.

## 2. The Study Motivation and Objectives

The study motivation of the intelligent roadways optical network based on the cloud edge terminal architecture mainly includes the following aspects:

(1) Meeting the demand for large-scale data processing: with the development of intelligent transportation systems, the amount of data that need to be processed is increasing. Traditional local computing methods cannot meet this demand. The intelligent roadways optical network based on the cloud-edge-terminal architecture can leverage the cooperative work of cloud computing, edge computing, and terminal devices to achieve large-scale data processing and analysis and improve data processing efficiency.
(2) Improving system real-time performance and response speed: The intelligent roadways optical network needs to transmit and process information such as vehicle location, speed, and traffic signals in real time to support traffic management and decision-making. The smart road optical network based on the cloud-edge-terminal architecture can leverage the advantages of cloud computing and edge computing to achieve fast data processing and response and improve the system’s real-time performance and response speed.
(3) Reducing network latency and costs: Traditional local computing methods require data to be transmitted to a central server for processing, resulting in large network latency and high costs. The intelligent roadways optical network based on the cloud-edge-terminal architecture can leverage edge computing and terminal devices for data processing, reducing data transmission latency and costs and improving the efficiency and scalability of the system.
(4) Promoting the development of intelligent transportation systems: The intelligent roadways optical network based on the cloud-edge-terminal architecture is an important component of intelligent transportation systems and can be interconnected with other intelligent facilities to achieve intelligentization and high-efficiency urban services. At the same time, this research can provide theoretical and technical support for the further development of intelligent transportation systems.

In summary, the study motivation of the intelligent roadways optical network based on the cloud-edge-terminal architecture is to meet the demand for large-scale data processing, improve system real-time performance and response speed, reduce network latency and costs, and promote the development of intelligent transportation systems.

The study objectives of the intelligent roadways optical network based on the cloud-edge-terminal architecture may include the following aspects:

(1) Building an efficient and reliable intelligent roadways optical network: to study how to leverage cloud computing, edge computing, and terminal devices to build an efficient and reliable smart road optical network that meets the requirements of large-scale data processing and real-time transmission.
(2) Enhancing data processing and analysis capabilities: to study how to leverage the advantages of cloud computing and edge computing to enhance data processing and analysis capabilities, achieve fast data processing and response, and provide more accurate and timely data support for traffic management and decision-making.
(3) Reducing network latency and costs: to study how to leverage edge computing and terminal devices for data processing, reduce data transmission latency and costs, improve system efficiency and scalability, and provide more economical and feasible solutions for the development of intelligent transportation systems.
(4) Promoting the development of intelligent transportation systems: to study how to integrate the intelligent roadways optical network based on the cloud-edge-terminal architecture with other intelligent facilities to achieve interconnection and interworking, realize the intelligence and efficiency of highway services, and promote the development of intelligent transportation systems.

All in all, the study objectives of the intelligent roadways optical network based on the cloud-edge-terminal architecture are to build an efficient and reliable intelligent roadways optical network, enhance data processing and analysis capabilities, reduce network latency and costs, and promote the development of intelligent transportation systems.

The methodological framework for researching the intelligent roadways optical network based on the cloud-edge-terminal architecture includes the steps of clarifying research objectives, collecting relevant materials, conducting demand analysis, designing the system, implementing and testing, performing data analysis and optimization, and obtaining user feedback and evaluation. Through the implementation of these steps, a highly efficient and reliable intelligent roadways optical network can be gradually constructed, and its performance and stability can be continuously improved.

## 3. The Overall Architecture of Fiber Optic Transmission Network for Cloud–Edge Cooperative

### 3.1. The Over-the-Horizon Scenario Division and Optical Fiber Communication

**Figure 1:**
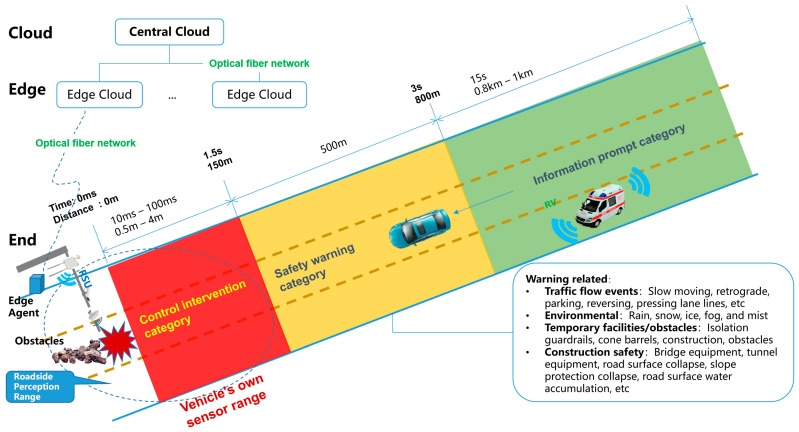
The overall architecture of fiber optic transmission network based on cloud–edge cooperative.

The cloud–edge collaborative architecture based on edge containers for vehicle-infrastructure cooperative autonomous driving is designed to accommodate the extensive needs of future intelligent high-speed or urban road collaborative autonomous driving. This architecture comprises end-side equipment, edge container cloud, and central cloud systems [5]. The overall architecture of the vehicle-infrastructure cooperative automatic driving system’s edge container cloud–cloud edge–collaborative cloud control platform includes a bottom-up step-by-step data processing and control instruction delivery logic. The overall architecture of a fiber optic transmission network based on the cloud–edge cooperative is illustrated in Figure 1. The three-stage segmentation method mainly solves the problem of dividing the video data collected from roadside digital infrastructure into red, yellow, and green areas according to specific rules for the beyond-visual range in intelligent road construction. It represents the control intervention area, safety warning area, and information prompt area, and can greatly simplify the complexity of roadside perception fusion algorithms and improve computational efficiency.

Based on the mechanism of vehicle dynamic distance envelope warning, when vehicles on highways pass through specific scenarios, according to the cloud control platform architecture and beyond the line-of-sight perception–distance division model of the vehicle-infrastructure–cooperative autonomous driving edge-container–cloud edge cooperative model, this vehicle will be in three alternating states during driving: information prompt type, safety warning type, and control intervention type [6]. Assuming that there is an obstacle at the deployment point of Class A equipment in the intelligent infrastructure of the expressway, after the roadside perception system recognizes the road obstacle, it will send different application scenario information to the vehicles in the way through the roadside intelligent terminal RSU (Roadside Unit, RSU) according to the distance from the obstacle. When the vehicle is 0.8–1 km away from the obstacle (it takes 15 s to drive), the roadside intelligent terminal mainly provides V2N (Vehicle to Network, V2N) services; when the vehicle is 0.15 km–0.5 km (1.5 s–3 s) away from the obstacle, the roadside intelligent terminal mainly provides V2I (Vehicle to Infrastructure, V2I)/I2V (Infrastructure to Vehicle, I2V) services. At this time, the information services will mainly be perceived, analyzed, and processed by the roadside infrastructure itself and provided to the passing vehicles; when the vehicle is within 0.15 km of the obstacle, due to the delay of roadside intelligent terminal information transmission, collection and processing, the roadside infrastructure is no longer able to provide effective information services within the limited time delay. It is necessary to approach the vehicle’s own perception and control intervention to ensure the safety of vehicle driving [7].

**Figure 2:**
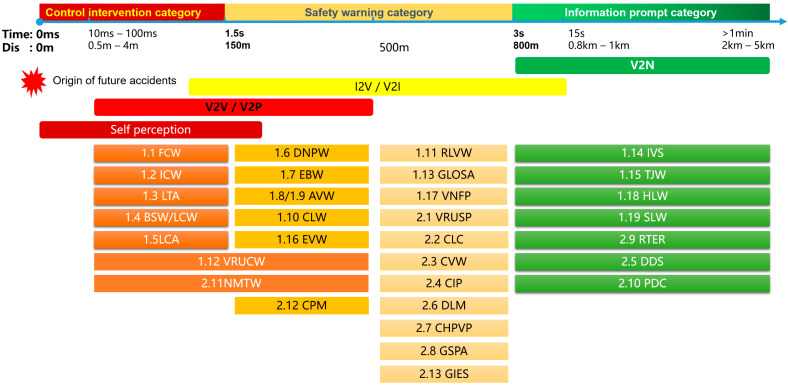
Vehicle-infrastructure cooperative intelligent driving cloud edge cooperative application scenario division.

According to the “Cooperative intelligent transportation system; vehicular communication; application layer specification and data exchange standard” (T/CSAE 53-2017) issued by the China Association of Automotive Engineers in 2017, 17 application scenarios are proposed, namely Forward Collision Warning (FCW), Intersection Collision Warning (ICW), Left Turn Assist (LTA), Blind Spot Warning/Lane Change Warning (BSW/LCW), Reverse Overtaking Warning (DNPW), Emergency Brake Warning (EBW), Abnormal Vehicle Warning (AVW), Control Loss Warning (CLW), Hazardous Location Warning (HLW), Speed Limit Warning (SLW), RLVW (Red Light Violation Warning), VRUCW (Vulnerable Road User Collision Warning), GLOSA (Green Light Optimal Speed Advisory), IVS (In Vehicle Signage), TJW (Traffic Jam Warning), EVW (Emergency Vehicle Warning), and Vehicle Field Payment (VNFP) [8]. The China Intelligent Transportation Industry Alliance issued the “Cooperative intelligent transportation system; vehicular communication; application layer specification and data exchange standard (Phase 2)” (T/ITS 0118-2020) in 2020, which mentions 12 application scenarios for Day 2, including Perceived Data Sharing (SDS), Collaborative Lane Change (CLC), Collaborative Vehicle Entry (CVM), Collaborative Intersection Traffic (CIP), Differential Data Service (DDS), Dynamic Lane Management (DLM), Collaborative Priority Vehicle Access (CHPVP), Station Path Guidance Service (GSPA), Floating Vehicle Data Collection (PDC), Vulnerable Traffic Participant Safe Access (VRUSP), Collaborative Vehicle Formation Management (CPM), and Road Toll Service (RTS) [9]. A cloud edge–collaborative cloud control platform and beyond-line-of-sight scene-partitioning model combines vehicle–road collaborative autonomous driving edge containers, after analyzing and integrating the three stages of the above application scenarios and beyond-the-line-of-sight scenarios, as shown in Figure 2.

According to the analysis of driver response characteristics in “Fundamentals of Traffic Engineering” [10], the minimum response time required by the driver before starting braking is 0.4 s, and it takes 0.3 s to produce a braking effect. In total, the minimum total time for braking effect is 0.7 s [11]. According to the regulations of the Association of State Highway Workers in the United States, the judgment time is 1.5 s, and the action time is 1 s. Therefore, the entire time from perception, judgment, starting braking to braking effectiveness is calculated as 2.5 to 3.0 s. The Table 1 is calculated based on 1.5 s.

**Figure 3:**
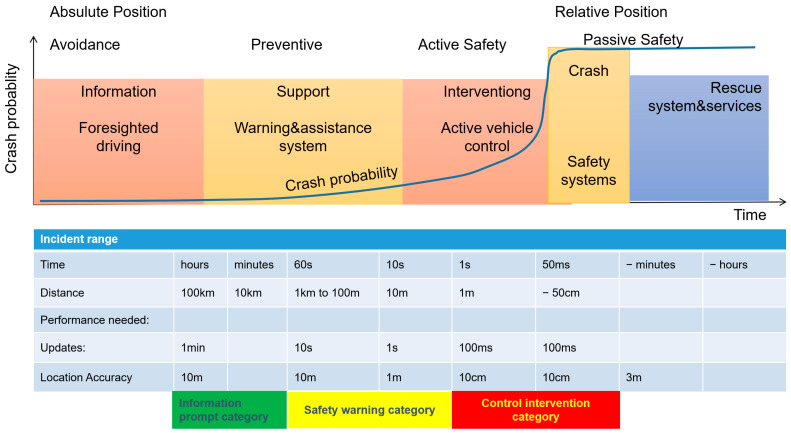
The analysis of the relationship between time, distance and positioning accuracy before and after traffic accidents.

Braking distance formula: S = V×V/2a;

Brake acceleration: 0.6 g–0.9 g;

Road surface condition: normal road friction coefficient;

The calculation of braking distance is shown in Table 1 and Figure 3.

Through the analysis in Figure 3, combined with the scene classification method in Figure 1, based on the different distances before and after the occurrence of a traffic accident, the information service types are divided into information prompt, safety warning and control intervention. This can guide roadside intelligent infrastructure to scientifically and efficiently serve passing vehicles, ensuring safer passage.

**Figure 4:**
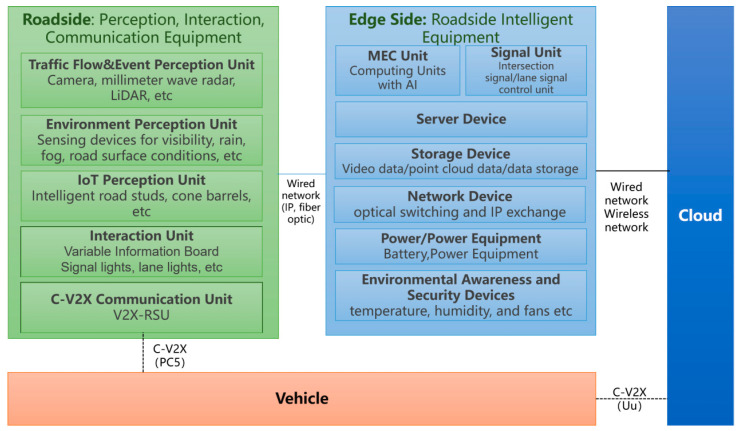
System architecture of end-side devices of vehicle-infrastructure cooperative automatic driving.

End-side equipment refers to the intelligent terminal equipment with a stable and reliable network connection, real-time network online and OTA upgrade on highways or urban roads, including but not limited to roadside intelligent terminal equipment and vehicle-end intelligent terminal equipment. The overall architecture is shown in Figure 4.

### 3.2. Optical Fiber Communication Based on Cloud Edge Architecture

**Figure 5:**
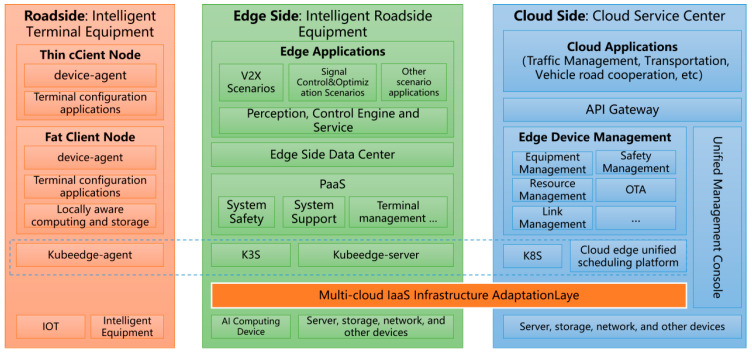
Overall architecture of cloud-side system for vehicle-infrastructure cooperative autonomous driving.

The edge cloud, represented by the edge container cloud, refers to equipment deployed on highways or urban roads with edge computing capabilities and computing power, which mainly includes two categories: an ECU (Edge Computing Unit, ECU) and a regional edge cloud control platform or intelligent intersection edge cloud control platform; in the highway scene, an ECU is deployed along the highway side according to the intelligent road level. In urban road scenarios, ECUs are deployed along urban roads or at intersections according to the level of intelligent intersections, and the regional edge cloud control platform or intelligent intersections edge cloud control platform can be referred to as an edge cloud, which is generally deployed in the data center room of regional road sections or urban road intersections according to the actual deployment environment of the project. An edge container cloud provides container-based digital cloud infrastructure in the form of a proprietary cloud/public cloud. Based on these infrastructures and virtualization technologies, combined with technical capabilities in network computing, storage, security and other aspects, a lightweight, high-performance, high-security edge collaborative control architecture system of an edge container can be formed. Through the opening of this architecture system capability and the combination of soft and hard, it provides a more convenient and exclusive edge container–cloud edge end collaborative control model (Figure 5) for upper-layer applications [12].

**Figure 6:**
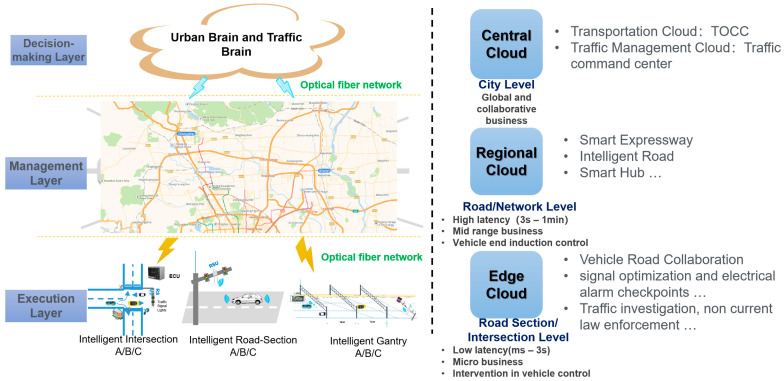
Business relationships of various clouds in the collaborative cloud control platform at the cloud side of the edge container of vehicle-infrastructure cooperative autonomous driving.

The central cloud system refers to the intelligent road operation control center for vehicle and road collaborative autonomous driving based on the cloud native technology architecture, that is, the cloud native cloud control center. According to the number of highways managed by the service or the number of urban road intelligent intersections, the road belongs to different administrative divisions; the cloud native cloud control center can be divided into two levels, respectively: a regional cloud–native cloud control center and a central cloud–native cloud control center [13]. In a central cloud–native cloud control center for city-level global and collaborative business, typical applications such as traffic TOCC (Transportation Operations Coordination Center, TOCC) or traffic control cloud of traffic command center, the main function is data-driven intelligent decision-making. The regional cloud–native cloud control center is oriented to road and road network applications, and the communication delay is between 3 s–1 min. The typical applications include intelligent high-speed, intelligent road, intelligent hub event recognition, path planning and vehicle end guidance and control, etc. The main function is data-driven intelligent management. The business relationships of various cloud–-native cloud control centers are shown in Figure 6.

**Figure 7:**
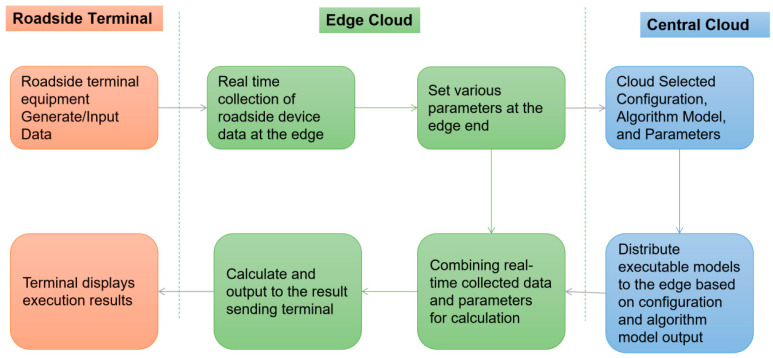
Cloud edge collaborative control model of edge container business process.

The cloud edge cooperative control model of an edge container adopts the distributed computing architecture, which transfers the computing of applications, data and services from the central node of the network to the logical edge node of the network. Edge computing will be completely handled by the central node to decompose large services, cut into smaller and more manageable parts, scattered to the edge node to deal with. The edge node is closer to the user terminal device, which can speed up the processing and transmission of data and reduce the delay (Figure 7).

**Figure 8:**
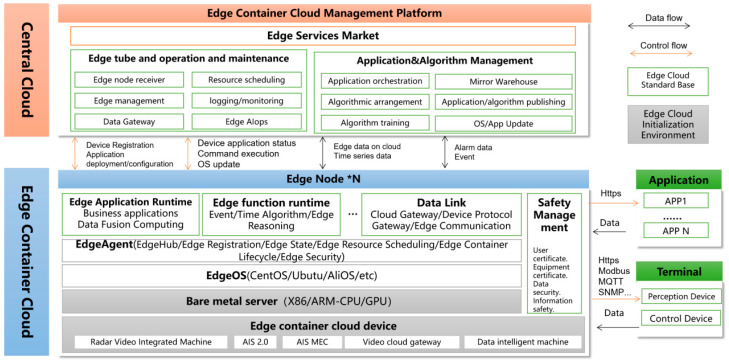
Intelligent road data flow and control flow diagram of vehicle-infrastructure cooperative autonomous driving.

Based on the overall architecture analysis of intelligent road logic for vehicle-infrastructure cooperative autonomous driving, the common elements in the link relationship between edge container cloud and central cloud are extracted at the level of cloud native cloud control center, and the data flow and control flow are sorted out and defined to form the data flow and control flow relationship of typical components of edge container cloud and central cloud, as shown in Figure 8.

**Figure 9:**
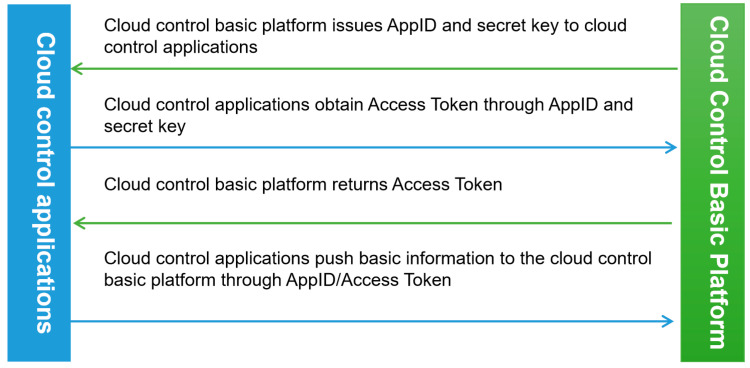
Cloud control platform data interaction protocol.

The cloud control basic platform issues the AppID (Application Programming Interface ID, AppID) and secret key (cloud control application registration key) to the cloud control application, and then uses the AppID and secret key to obtain the Token (application registration credentials) from the cloud control basic platform authentication server. Finally, the data interaction is completed through the AppID and Token using RESTful API based on the HTTPS application layer protocol and JSON data format. The blue arrow represents the request sent by the cloud control application to the cloud control basic platform. The green arrow represents the request response of the cloud control basic platform to the cloud control application. The specific interaction process is shown in Figure 9.

### 3.3. Interactive Multiple-Model Algorithm for Highway Vehicle Target Tracking

1. Interactive multiple model Kalman filtering algorithm

IMMKF (Interactive Multiple Model Adaptive Kalman Filter, IMMKF) adopts several motion models for the movement of target vehicles on highways and adopts a probability conversion mechanism between these motion models. It is achieved through multi-channel parallel filtering, and each filter conforms to the multi-channel model. Due to the mutual conversion between different models, there is also some information exchange between filters. During each sampling period, it is highly likely that all filters in IMMKF are in operation. The overall state estimation is formed by merging the state estimates of various filters. M_1_, M_2_, M_3_, …, M_i_ are the i models of IMMKF, where M_r_(*k*) indicates that the model M_r_ is valid during the sampling period and ends at frame *k*. X represents the target state, and X(*k* + 1) represents the target state value at frame *k* + 1. During the occurrence of time M_i_(*k* + 1), *w*(*k*) and v(*k*) represent the variable value of the state estimation value of the target state at frame *k*; the development of the target state follows the following formula:

(1) $$X(k+1)=F_{i}X(k)+w_{i}(k)$$

The measurement formula is:

(2) $$z(k+1)=H_{i}X(k+1)+v_{i}(k+1)$$

**Figure 10:**
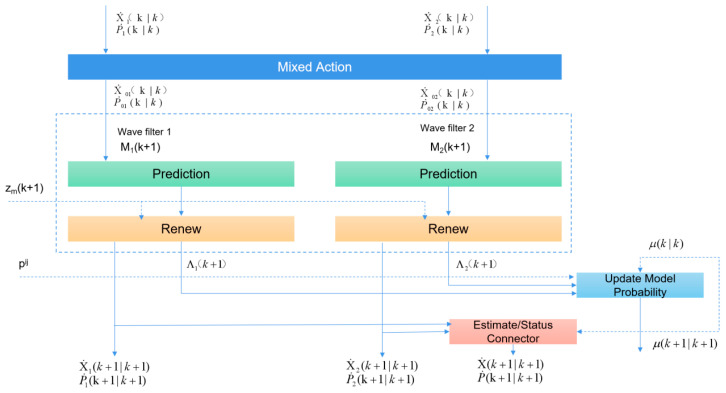
One-loop operation of interactive multiple model IMMKF.

The flowchart of the interactive multi-model IMMKF iteration loop is shown in Figure 10.

2. Interaction and Mixing

For the event M*_i_*(*k* + 1), the calculation process of mixed estimation X_0_*_i_*(*k*|*k*) and covariance matrix P_0_*_i_*(*k*|*k*) is as follows:

$$\begin{array}{l} {\hat{X}}_{0j}(k|k)=\sum_{i=1}^{r}\mu_{i|j}(k|k){\hat{X}}_{i}(k|k)\\ {\hat{P}}_{0j}(k|k)=\sum_{i=1}^{r}\mu_{i|j}(k|k)\left\{{\hat{P}}_{i}(k|k)+[{\hat{X}}_{i}(k|k)-{\hat{X}}_{0j}(k|k)]\right\} \end{array}$$

The mixed probability $\mu_{i|j}(k|k)$ is

$$\mu_{i|j}(k|k)=\frac{1}{\mu_{j}(k+1|k)}P_{\mathrm{ij}}\mu_{i}(k|k)$$

The calculation process of the prior prediction mode probability $\mu_{j}(k+1|k)$ is as follows:

$$\mu_{j}(k+1|k)=\sum_{i=1}^{r}p_{ij}\mu_{i}(k|k)$$

Model transformation is achieved through the model process and determined by the following model transition probabilities

$$P_{\mathrm{ij}}=P_{r}\left\{M_{j}(k+1)|M_{i}(k)\right\}$$

where $P_{r}\left\{•\right\}$ represents the probability of the event occurring; in other words, *P*_ij_ is the probability of the *M_i_* model at time *k* being converted to the *M_j_* model at time *k* + 1, which can be used to calculate the final output model probability.

3. Kalman filtering

As shown in Figure 10, the usual KF equation is used to handle appropriate highway target vehicle movement models and update the mixed state estimation with the current measurement values, with a new covariance of:

(3) $$S_{j}=H_{j}{\tilde{P}}_{j}(k+1|k)H^{T}{}_{j}+R_{j}$$

The new sequence can be calculated using the following equation:

$$\nu_{j}=z(k+1)-{\tilde{z}}_{j}(k+1|k)$$

The matched filter remembers that the likelihood function is a Gaussian probability density function about information *v_j_*, with a mean of 0 and a covariance of *S_j_*. This can be used to update the probabilities of various different models, and the calculation process is as follows:

$$\Lambda j=\frac{1}{(2\prod {)}^{0.5n}\sqrt{\left|S_{j}\right|}}\exp\left\{-0.5\nu^{T}{}_{j}S^{-1}{}_{j}\nu_{j}\right\}$$

where n is the dimension of the information vector *v*.

4. Model probability update

As long as the model obtains the data update according to the measured value z(*k* + 1), the model likelihood function $\Lambda_{j}$ is used to update the model probability $\mu_{j}(k+1|k+1)$. Then, the prediction statistical model $\mu_{j}(k+1|k)$ of M_j_(*k* + 1) is:

$$\mu_{j}(k+1|k+1)=\frac{1}{c}\mu_{j}(k+1|k)\Lambda_{j}$$

Among them, the normalization factor is:

$$c=\sum_{i=1}^{r}\mu_{i}(k+1|k)\Lambda_{i}$$

Combiner of state estimation and covariance.

By updating the model probability $\mu_{i}(k+1|k+1)$, the estimated state ${\hat{X}}_{j}(k+1|k+1)$ and covariance $P_{j}(k+1|k+1)$ of each filter are merged to obtain the overall state estimation $\hat{X}(k+1|k+1)$ and corresponding covariance $\hat{P}(k+1|k+1)$. The calculation process is as follows:

$$\hat{X}(k+1|k+1)=\sum_{j=1}^{r}\mu_{j}(k+1|k+1){\hat{X}}_{j}(k+1|k+1)$$

$$P(k+1|k+1)=\sum_{j=1}^{r}\mu_{j}(k+1|k+1)\{P_{j}(k+1|k+1)+[X_{j}(k+1|k+1)\}-\;\;\;\;\;\;\;\;\;\;\;\hat{X}(k+1|k+1)][{\hat{X}}_{j}(k+1)-\hat{X}(k+1|k+1){]}^{T}\left.\right\}$$

Target Movement Model of Highway Vehicles

The two common models for target movement of highway vehicles are the 2-DOF (Degree of Freedom, DOF) dynamic model, also known as the constant velocity model, and the other is the 3-degree of freedom dynamic model, also known as the constant acceleration model. This article will focus on explaining the constant acceleration model.

The position, velocity and acceleration components on the x, y and z axes of the 3-DOF dynamic model, also known as the constant acceleration model in the industry, are known. Its stochastic matrix and process noise matrix are as follows:

$$F_{CA}=\left[\begin{array}{ccc} \varphi_{CA} & 0 & 0\\ 0 & \varphi_{CA} & 0\\ 0 & 0 & \varphi_{CA} \end{array}\right]\;G_{CA}=\left[\begin{array}{ccc} \varsigma_{CA} & 0 & 0\\ 0 & \varsigma_{CA} & 0\\ 0 & 0 & \varsigma_{CA} \end{array}\right]$$

wherein

$$\varphi_{CA}\left[\begin{array}{ccc} 1 & T & T^{2}/2\\ 0 & 1 & T\\ 0 & 0 & 1 \end{array}\right]\;\varsigma_{CA}=\left[\begin{array}{c} T^{{}_{3}}/6\\ F^{2}/2\\ T \end{array}\right]$$

The acceleration increment here follows the discrete time zero mean Gaussian white noise, and there is also a lower process noise covariance $\Omega_{CA}$, which can generate approximately uniform acceleration motion. Assuming that the covariance of process noise on each coordinate is equal ($\sigma^{2}{}_{x}=\sigma^{2}{}_{y}=\sigma^{2}{}_{z}$). Through extensive research and development, the use of higher process noise in 3-degree of freedom dynamic models can track the beginning and end stages of highway vehicle targets within a certain range.

### 3.4. Intelligent Highway Vehicle Target Detection

**Figure 11:**
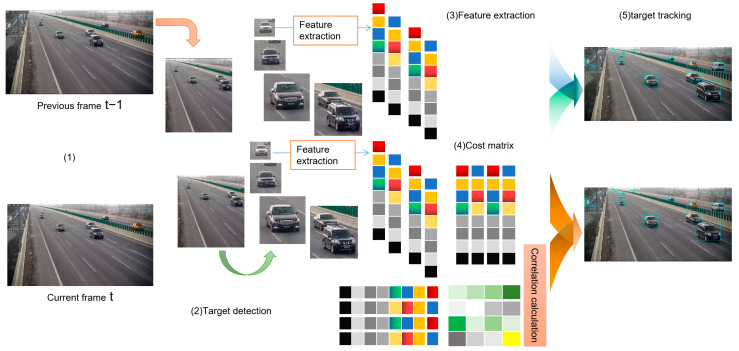
A real time universal detection model based on StreamYOLO.

A real-time universal detection model based on StreamYOLO, supporting eight types of traffic object detection. StreamYOLO is based on the YOLOX model and uses a Dual Flow Perception feature fusion module to learn temporal relationships at the feature level, improving the ability to predict environmental perception. At the same time, StreamYOLO designed a Trend Aware Loss to perceive the intensity of object motion changes, which is used to weight the regression of object prediction, enabling objects with drastic changes in motion to obtain higher regression weights and better prediction results. Step (1) is to extract target recognition image frame data. Step (2) is to detect targets in image frames. Step (3) is to extract target features. Step (4) is to perform relevant calculations using the cost matrix algorithm. Step (5) is to output target tracking data in continuous images. The real-time universal detection model for highway vehicle video detection based on StreamYOLO is shown in Figure 11.

This article has been optimized through experiments in the field of StreamYOLO real-time video object detection and autonomous driving in the open-source large model website ModelScope [14]. Based on the StreamYOLO model mentioned above, this article provides 3 s of real-time video-stream data from highway roadside perception. It is tested and verified on the large model website ModelScope. After importing the model, the inference time for the test model is 10.6 s, and the results are displayed.

**Figure 12:**
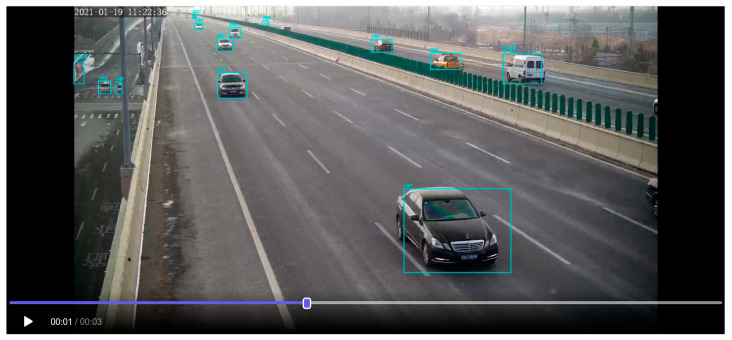
Test results of highway roadside perception video stream data model.

According to the beyond-line-of-sight perception distance division method, known as the “three segment region division method”, the intelligent camera’s video range is color-coded based on the three segment regions. Through precise positioning algorithms like histogram analysis, Zhang’s correction method, the least squares method, and affine transformation, lane lines and road isolation barriers are accurately identified. Additionally, algorithms like Yolo object detection, Kalman filtering, and others are combined with SORT target tracking and the Hungarian algorithm to annotate information on vehicle boxes and corresponding speeds. This annotation assigns three different colors to vehicle boxes and corresponding speed information in different regions. The results are summarized in Figure 12.

## 4. Intelligent Highway Deployment Architecture

### 4.1. Intelligent Highway Deployment Architecture

**Figure 13:**
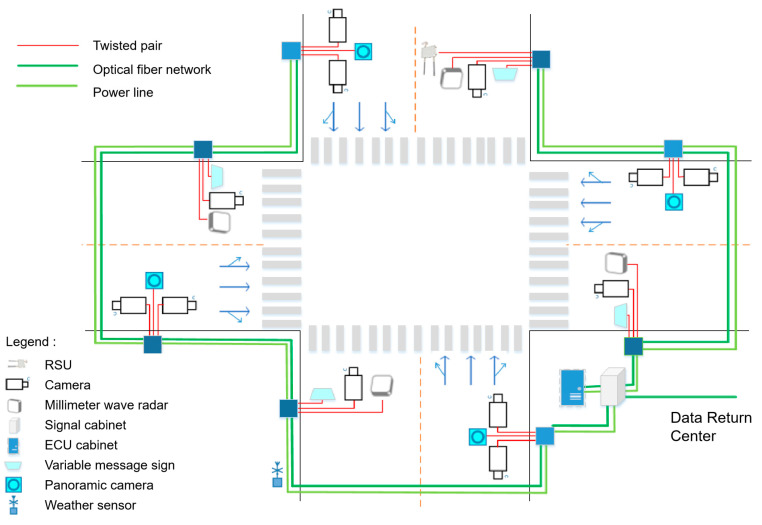
Physical architecture diagram of intelligent intersection fiber optic network composition.

The intelligent road physical architecture is a description of the physical entity deployment relationship after analyzing the service object, service content and logical architecture elements. The intelligent road will build a roadside traffic infrastructure integrating perception, calculation, control, optical fiber network communication, interaction, management and service on the roadside [15]. The physical architecture diagram of intelligent intersection fiber optic network composition is illustrated in Figure 13.

As shown in Figure 13, in the hardware deployment of smart intersections, roadside digital infrastructure such as RSU, cameras, millimeter wave radar, signal cabinets, ECU cabinets, variable message cameras, panoramic cameras, and weather stations are deployed in different directions of the intersection. If multiple poles are combined, the installation poles of the electronic police can be shared. When these numerous sensors are deployed at intersections, in addition to providing power to each device, they also need to be connected through fiber optic networks to form a local area network and provide high computing power and speed computing services at the edge. The green connecting line in Figure 13 represents the fiber optic network.

### 4.2. Demonstration of Highway Deployment Effect

**Figure 14:**
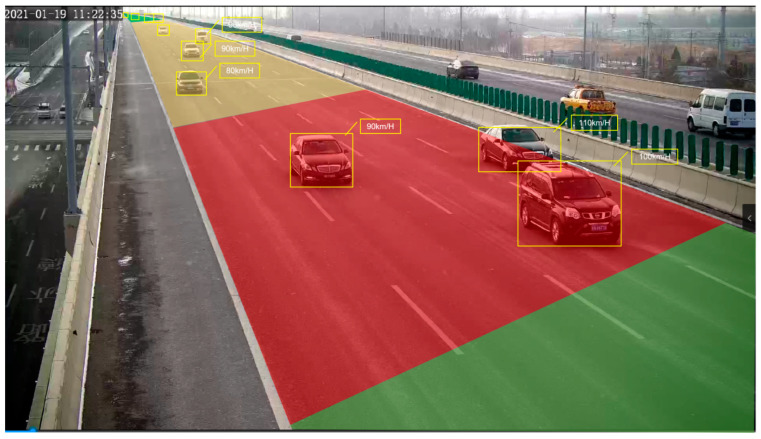
Intelligent infrastructure for vehicle recognition, tracking and cooperative control based on over-the-horizon scene division.

The intelligent infrastructure deployed on highways automatically recognizes the lane lines of highways based on the over-the-horizon scene division model and annotates the videos collected by intelligent cameras with different colors according to the three scene division methods. Video analysis algorithms and vehicle dynamic envelope warning technology are used to identify the size, speed, driving direction, and status of vehicles in real time, to conduct real-time tracking and analysis of vehicles passing through this area, and to provide cloud edge application services in three different states [16]. The intelligent infrastructure for vehicle recognition, tracking and cooperative control based on over-the-horizon scene partitioning is shown in Figure 14.

In the experiment after actual deployment on highways, it is necessary to identify and judge the driving trajectory of vehicles in the video and predict the driving path of each vehicle within the video range in advance [17].

According to the beyond line of sight perception distance division method, known as the “three segment region division method,” that consists of information category, safety warning category, and control intervention category, the intelligent camera’s video range is color-coded based on the three segment regions. If traffic accidents are divided based on distance or time before and after their occurrence, they are divided into information prompt categories—green colors. The cloud control platform provides V2N beyond line of sight roadside perception information services to traffic participants based on the perception data of roadside perception devices. For safety warning category-yellow colors, after the roadside perception equipment perceives the traffic accident information, it is submitted to the nearest edge computing unit for fusion processing, and then provided to the roadside intelligent terminal RSU through the roadside switch to provide the traffic participants with an I2V roadside perception information service. In terms of control interventions category—red colors, due to the fast driving speed and short time of vehicles on highways, coupled with the current lack of standardized collaborative control for vehicle operation through roadside digital infrastructure, roadside digital infrastructure is no longer capable of providing I2V collaborative control information for vehicle decision-making within a limited time delay range, and can only rely on the perception of both vehicles’ decision-making and control equipment to achieve safe driving.

## 5. Conclusions

This article provides an overview and analysis of current research progress, project status, and main issues in the fields of over-the-horizon perception and cooperation control. Through analysis of roadside cloud edge and vehicle-infrastructure cooperation perception control and scene analysis, a classification of specific scenarios on highways and the corresponding “three-segment regional division method” are proposed. Specifically, within the field of view of roadside sensors’ intelligent cameras, the rectangular region composed of the driving lane is divided into three areas: information prompt area, safety warning area, and control intervention area. These three areas are marked with green, yellow, and red in real-time videos. When vehicles on highways pass through these three areas, the system will select which type of roadside information service to provide to traffic participants based on the target vehicle’s current location, speed, lane, etc., ensuring that they can enjoy over-the-horizon perception services and I2V vehicle–road collaborative services provided by roadside perception devices.

In addition, through the interactive multi-model algorithm for target vehicle detection and tracking in vehicle-infrastructure operating scenarios, the method of local trajectory construction for vehicle-side perception of roadside perception, and the implementation of real-time video for target vehicle detection and tracking on the roadside, further verification of the three-segment regional division method combines the scene fusion division released by DAY1 and DAY2. This will also provide a relatively intuitive and quantifiable technical approach for roadside sensor fusion perception to provide which type of information to vehicles. This approach will form a reference for the cooperation development of vehicle-infrastructure and intelligent connected vehicles in the Chinese solution for intelligent driving in vehicle-infrastructure cooperation systems.

## Figures and Tables

**Table 1:** Calculation of braking distance.

No	Starting Speed (km/h)	Warning	Emergency								
		Within the Notification Time (s), the Distance Traveled (m)	Distance Traveled (m) within the Judgment + Action Time (s)			Under the Specified Braking Acceleration (g), the Distance Traveled from Applying the Brake to Stopping (m)			Distance Traveled from Problem Discovery to Parking (m)		
		15 s	0.7 s	1.5 s	2.5 s	0.9 g	0.8 g	0.6 g	Min- Distance	General Distance	Max-Distance
1	20	83.33	3.89	8.33	13.89	1.75	1.97	2.62	5.64	10.30	16.51
2	40	166.67	7.78	16.67	27.78	7.00	7.87	10.50	14.78	24.54	38.28
3	60	250.00	11.67	25.00	41.67	15.75	17.72	23.62	27.41	42.72	65.29
4	80	333.33	15.56	33.33	55.56	27.99	31.49	41.99	43.55	64.83	97.55
5	100	416.67	19.44	41.67	69.44	43.74	49.21	65.61	63.19	90.88	135.06
6	120	500.00	23.33	50.00	83.33	62.99	70.86	94.48	86.32	120.86	177.82
7	140	583.33	27.22	58.30	97.22	85.73	96.45	128.60	112.96	154.78	225.82
8	160	666.67	31.11	66.67	111.11	111.98	125.98	167.97	143.09	192.64	279.08
9	180	750.00	35.00	75.00	125.00	141.72	159.44	212.59	176.72	234.44	337.59

## Data Availability

The data used to support the findings of this study are included within the article.
